# Association of variant in the ADIPOQ gene and functional study for its role in atherosclerosis

**DOI:** 10.18632/oncotarget.21232

**Published:** 2017-09-23

**Authors:** Xinzhong Chen, Yanhong Yuan, Yufeng Gao, Qin Wang, Fei Xie, Dongsheng Xia, Yutao Wei, Ting Xie

**Affiliations:** ^1^ Department of Cardiovascular Surgery, Union Hospital, Tongji Medical College, Huazhong University of Science and Technology, Wuhan, 430022, China; ^2^ Department of Neurology, The Fifth Affiliated Hospital of Zhengzhou University, Zhengzhou, 450052, China; ^3^ Department of Cardiovascular Surgery, Henan Provincial People’s Hospital, Zhengzhou, 450003, China; ^4^ Department of Cardiac Surgery, Hainan Provincial People’s Hospital, Hainan 570311, China

**Keywords:** ADIPOQ, adiponectin, atherosclerosis, polymorphism

## Abstract

The burden of atherosclerosis is heritable and associated with elevated risk of developing CVDs. Here, we evaluated genetic variants of adiponectin (ADIPOQ) gene, which has important role in anti- atherosclerosis, with risk of atherosclerosis among a large Chinese population. Our results show that rs74577862 was significantly associated with risk of atherosclerosis (OR=2.08; 95%CI=1.48-2.91; P=2.2×10^-5^). When stratified by atherosclerosis site, rs74577862 was associated with increased risk of both carotid atherosclerosis (OR=2.03; 95%CI=1.35-3.06; P=6.3×10-4) and coronary atherosclerosis (OR=2.11; 95%CI=1.44-3.09; P=1.1×10-4). In addition, we also carried out site-directed mutagenesis and dual-luciferase reporter assay to confirm the positive finding, which presents a significant decrease in luciferase expression for the reconstructed plasmid with rs74577862 A allele in comparison to the one with G allele (P<0.001). Real-time PCR also confirmed the findings above. These results strongly suggest that the functional SNP, ADIPOQ rs74577862 might contribute to atherosclerosis susceptibility.

## INTRODUCTION

Cardiovascular disease (CVD), which refers a class of diseases that involve the heart or blood vessels, is the leading cause of death globally, and a major determinant of global health [1, 2]. It was estimated that CVD accounted for an estimated 17. 3 million (95% UI, 16.5–18.1 million) of 54 million total deaths, or 31.5% (95% UI, 30.3%–32.9%) of all global deaths [3]. While, the burden of atherosclerosis is heritable and associated with elevated risk of developing CVDs [4–8]. Up to 90% of atherosclerosis may be preventable with a proper screening program, while biomarkers identified from genetic association studies, including genome-wide association studies (GWAS), have provided us an efficacious approach for screening of atherosclerosis [9–16].

Adiponectin (ADIPOQ), an adipokine with anti-inflammatory, antioxidant, antiatherogenic, pro-angiogenic, vasoprotective and insulin-sensitizing properties, has been identified to be inversely associated with higher risk of atherosclerosis and could be used as a predictor [17–19]. This is because ADIPOQ may protected the aorta from atherosclerosis injury by reducing the oxidative stress, as well as reducing the lesion formation size in the aortic root and reducing TC, TG, and LDL-C in serum [20]. Recently, the UK10K project identified that a low-frequency intronic variant in ADIPOQ was associated with decreased ADIPOQ levels (rs74577862-A, effect allele frequency (EAF) = 2.6%, P value = 3.04 × 10^-64^) in participants of European ancestry [21]. However, whether ADIPOQ variants were associated with risk of atherosclerosis, especially in a non- European population, is still unknown. Here, we aim to evaluate the association of the genetic variants of ADIPOQ gene with risk of atherosclerosis among a large Chinese population with a case-control study design. Besides, we also selected tagSNPs of the ADIPOQ gene using the RegulomeDB score system and SNAP [22, 23], thus, we got rs74577862 and rs62292784 genotyped and included in our analyses. Furthermore, we also conducted functional study of ADIPOQ rs74577862 for its role in the development of atherosclerosis.

## RESULTS

### Characteristics of study participants

The characteristics of the study participants were summarized in Table 1. No significant differences were found between cases and controls for age group, gender, smoking status, and drinking status. For the atherosclerosis subtypes, we included 400 carotid atherosclerosis cases (44.4%) and 500 coronary atherosclerosis cases (55.6%) in this case-control study.

**Table 1:** Clinical and demographic characteristics of atherosclerosis cases and controls

Variables	Cases (n=900)	Controls (n=900)	P value
Age			
≥60	424 (47.1%)	411 (45.7%)	0.539
<60	476 (52.9%)	489 (54.3%)	
Gender			
Male	650 (72.2%)	634 (70.4%)	0.404
female	250 (27.8%)	266 (29.6%)	
Smoking status			
Smokers	280 (31.1%)	253 (28.1%)	0.055
Non-Smokers	620 (68.9%)	657 (71.9%)	
Alcohol status			
drinkers	292 (32.4%)	277 (30.8%)	0.447
Non-drinkers	608 (67.6%)	623 (69.2%)	
Subtypes			
Carotid atherosclerosis	400 (44.4%)		
Coronary atherosclerosis	500 (55.6%)		

### Association analysis between genetic variants of ADIPOQ gene with risk of atherosclerosis

The distribution of ADIPOQ rs74577862 and rs62292784 in atherosclerosis patient and control groups are presented in Table 2. The minor allele frequencies (MAF) were consistent with those in CHB of 1000 genomes data. Both of the two SNPs were in Hardy–Weinberg equilibrium in control group (P > 0.05). We found rs74577862 was significantly associated with risk of atherosclerosis (OR=2.08; 95%CI=1.48-2.91; P=2.2×10^-5^). Using QUANTO [32], we have a 99.2% power for rs74577862 in current analysis. Compared with carriers of genotype GG, those with genotype AG (OR=1.94; 95%CI=1.34-2.80; P=4.0×10^-4^) and AA (OR=4.38; 95%CI=1.08-17.7; P=0.038) have significantly increased risk of atherosclerosis. In dominant model, rs74577862 was also associated with increased risk of atherosclerosis (OR=2.03; 95%CI=1.43-2.90; P=8.5×10^-5^). However, we didn’t find any significant associations for rs62292784.

**Table 2:** Associations of genetic variants of ADIPOQ with atherosclerosis risk

	atherosclerosis cases	Controls	OR (95% CIs)^*^	P value
rs74577862				
GG	807 (89.7%)	850 (94.4%)	1.00 (Reference)	
AG	85 (9.4%)	48 (5.3%)	1.94 (1.34-2.80)	**4.0×10^-4^**
AA	8 (0.9%)	2 (0.2%)	4.38 (1.08-17.7)	**0.038**
AA+AG	93 (10.3%)	50 (5.5%)	2.03 (1.43-2.90)	**8.5×10^-5^**
A vs G			2.08 (1.48-2.91)	**2.2×10^-5^**
MAF	0.056	0.029		
MAF in CHB of 1000 genomes		0.024		
rs62292784				
CC	338 (37.6%)	370 (41.1%)	1.00 (Reference)	
CT	452 (50.2%)	429 (47.6%)	1.20 (0.94-1.53)	0.138
TT	110 (12.2%)	101 (11.2%)	1.24 (0.87-1.77)	0.240
T vs C			1.15 (0.96-1.38)	0.136
MAF	0.373	0.351		
MAF in CHB of 1000 genomes		0.354		

* Adjusted for age, gender, smoking status, and drinking status.

MAF=minor allele frequency.

Multiple testing were conducted using Bonferroni correction, and 0.025 was used as the p value threshold for the two variants.

### Stratified analyses between ADIPOQ rs74577862 with risk of atherosclerosis

To find the potential population stratification, we analyzed the associations between ADIPOQ rs74577862 with risk of atherosclerosis stratified by atherosclerosis site. As shown in Table 3, we found rs74577862 was associated with increased risk of either carotid atherosclerosis (OR=2.03; 95%CI=1.35-3.06; P=6.3×10^-4^) or coronary atherosclerosis (OR=2.11; 95%CI=1.44-3.09; P=1.1×10^-4^).

**Table 3:** Associations of rs74577862 with atherosclerosis risk

	atherosclerosis cases	Controls	OR (95% CIs) ^*^	P value
Carotid atherosclerosis				
GG	359 (89.8%)	850 (94.4%)	1.00 (Reference)	
AG	38 (9.5%)	48 (5.3%)	1.95 (1.25-3.03)	**3.1×10^-3^**
AA	3 (0.7%)	2 (0.2%)	3.69 (0.71-19.3)	0.121
AA+AG	41 (10.3%)	50 (5.5%)	2.02 (1.31-3.10)	**1.3×10^-3^**
A vs G			2.03 (1.35-3.06)	**6.3×10^-4^**
Coronary atherosclerosis				
GG	448 (89.6%)	850 (94.4%)	1.00 (Reference)	
AG	47 (9.4%)	48 (5.3%)	1.93 (1.27-2.94)	**2.0×10^-3^**
AA	5 (1.0%)	2 (0.2%)	4.93 (1.15-21.2)	**0.032**
AA+AG	52 (10.4%)	50 (5.5%)	2.05 (1.37-3.07)	**4.6×10^-4^**
A vs G			2.11 (1.44-3.09)	**1.1×10^-4^**

* Adjusted for age, gender, smoking status, and drinking status.

Multiple testing were conducted using Bonferroni correction, and 0.025 was used as the p value threshold for the two variants.

### Site-directed mutagenesis and dual-luciferase reporter assay

**Figure 1:**
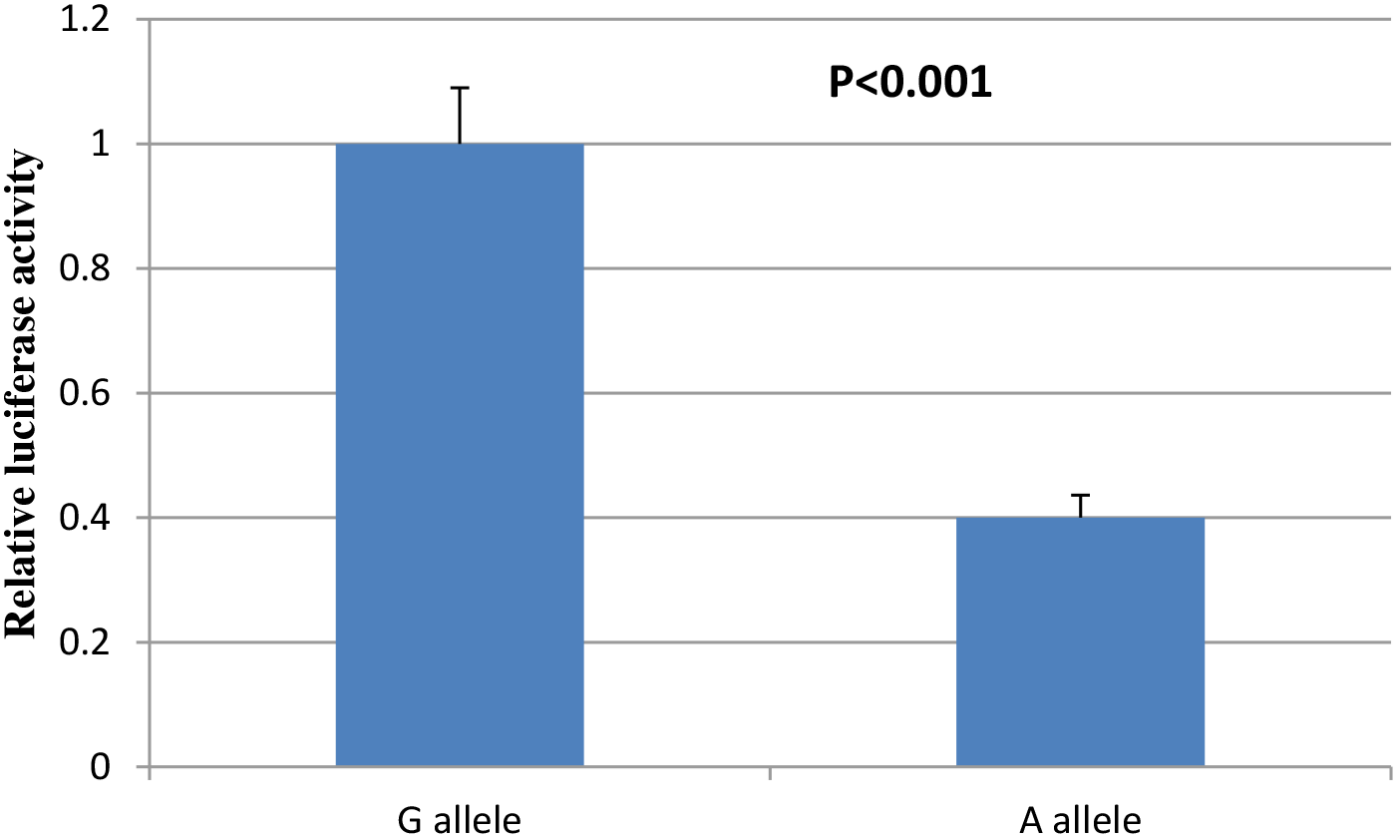
Dual-luciferase reporter assay results of ADIPOQ rs74577862

To evaluated the potential functions of ADIPOQ rs74577862, we conducted the Site-Directed Mutagenesis and dual-luciferase reporter assay. As shown in Figure 1, we found that a significant decrease in luciferase expression for the reconstructed plasmid with rs74577862 A allele in comparison to the one with G allele in HAECs cell lines (P<0.001). This means allele A was associated with lower level ADIPOQ, then increased risk of atherosclerosis.

### Quantification of gene expression using real-time PCR

**Figure 2:**
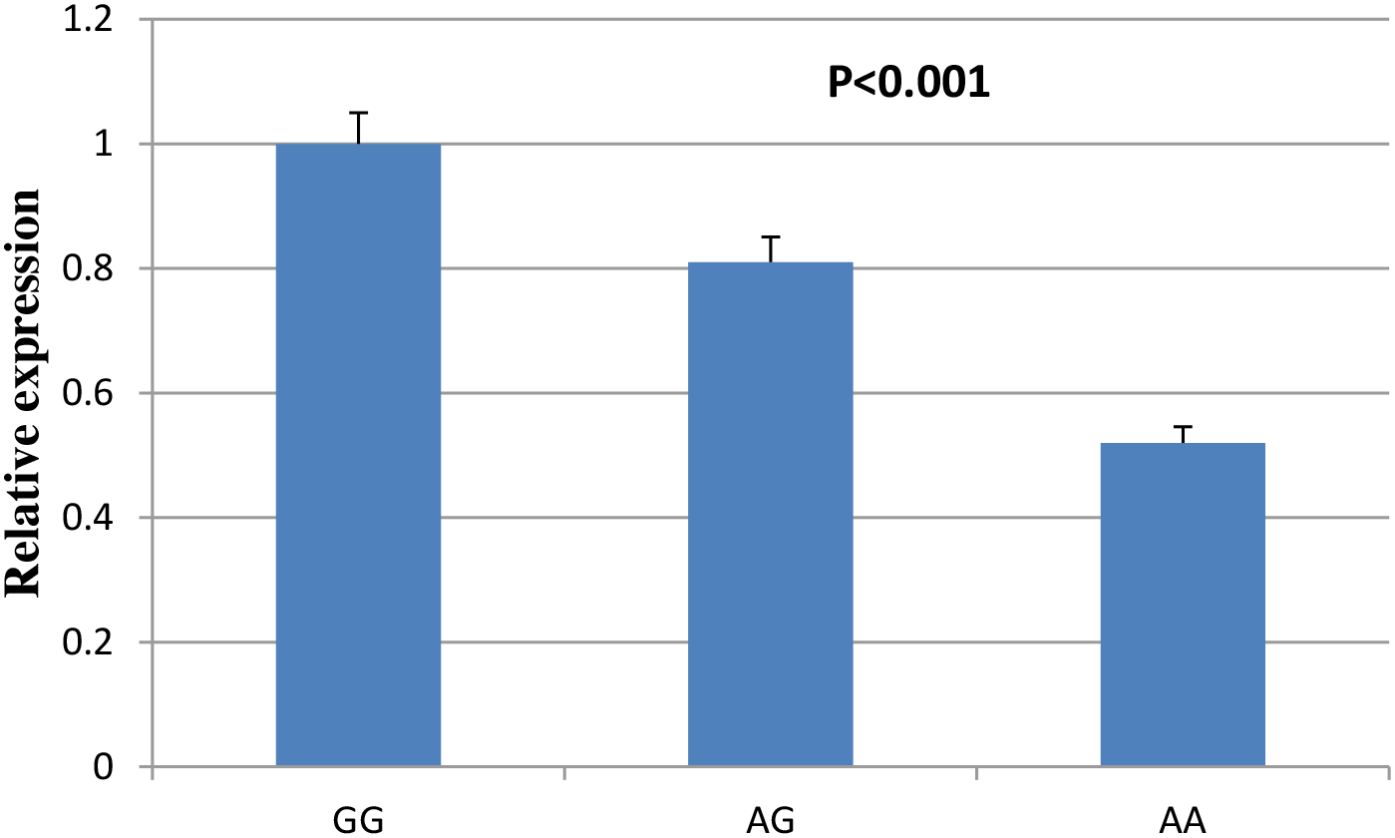
Comparison of ADIPOQ gene expressions between rs74577862 genotypes

We then assessed the influence of ADIPOQ rs74577862 on the expression of ADIPOQ gene in the plaque tissues of 50 individuals was confirmed by real time PCR. As shown in Figure 2, the atherosclerosis risk allele of rs74577862 correlated with decreased expression level of ADIPOQ gene (P<0.001).

## DISCUSSION

The current study explored association between genetic variants of ADIPOQ gene (rs74577862 and rs62292784) with risk of atherosclerosis among a large Chinese population. Significantly association with risk of atherosclerosis was detected for ADIPOQ rs74577862. Even stratified by atherosclerosis site, rs74577862 was still associated with increased risk of either carotid or coronary atherosclerosis. Then, we also conducted dual-luciferase reporter assay and real-time PCR of ADIPOQ rs74577862 for its role in the development of atherosclerosis.

It has been demonstrated that ADIPOQ worked as a novel modulator for endothelial adhesion molecules, and plasma ADIPOQ concentrations were significantly lower in patients with coronary artery disease early in 1999 [24]. ADIPOQ, which accumulated in the injured artery from the plasma and suppressed endothelial inflammatory response and vascular smooth muscle cell proliferation, could also suppress the development of atherosclerosis *in vivo* [25]. Epidemiological study also suggested that low level of plasma ADIPOQ was associated with higher risk of atherosclerosis, diabetes, insulin resistance, obesity, and cancers [26–29]. Inamura et al [30] reported that Low-level plasma adiponectin was associated with KRAS-mutant colorectal cancer risk.

SNP rs74577862 is located at 3q27.3, the intron region of ADIPOQ. We found that a significant decrease in luciferase expression for the reconstructed plasmid with rs74577862 A allele in comparison to the one with G allele, which was mutually confirmed with the previous finding that A allele of rs74577862 was associated with decreased ADIPOQ levels [21]. Using HaploReg v4.1 [31], we found rs74577862 could cause the change of motifs NF-kappaB and TATA. NF-κB signaling has been linked with lipid metabolism and atherosclerosis, and inhibition of NF-κB signaling has been shown to protect against atherosclerosis [32].

Our study had its own advantages. First, our large sample size warranted the liability of the results. Although as a low frequency variant, we still have a 99.2% power for rs74577862 in current study using QUANTO [33]. Second, we characterized the function of SNP rs74577862, making the association of this SNP with the risk of atherosclerosis biological plausible. Despite of these strengths mentioned above, some limitations should be noted. Firstly, lack of evaluation of gene–environment interactions; second, an independent replication. However, our results still provided us an important clue for the prevention of atherosclerosis. Future studies should address the mechanisms of ADIPOQ with regard to the risk of atherosclerosis.

In summary, the current study investigated the association between genetic variants of ADIPOQ gene and risk of atherosclerosis in a Chinese population. We identified ADIPOQ rs74577862 minor allele associated with a higher atherosclerotic burden. This polymorphism was also shown to be associated with increased risk of either carotid or coronary atherosclerosis, suggesting that rs74577862 has the similar involvements in the pathophysiology of carotid and coronary atherosclerosis. These findings provide further evidence for an important causal role of rs74577862 in atherosclerosis development.

## MATERIALS AND METHODS

### Study subjects

Totally included in current study were 900 atherosclerosis and 900 matched healthy controls. Clinical diagnosis of 500 coronary atherosclerosis cases was evaluated by percutaneous coronary angiography (a stenosis degree greater than or equal to 50% in at least one artery), confirmed by two experienced cardiologists. While 400 carotid atherosclerosis cases were defined as patients with evidence of carotid plaques (CPs) presence in the internal carotid artery (ICA) or the common carotid artery (CCA) who were consecutively admitted for carotid endarterectomy (greater than or equal to 70% NASCET stenosis). A complete medical history together with demographic characteristics was compiled for each individual enrolled by a face to face interview in the study. The exclusion criteria for all patients were those with systemic diseases such as inflammation, tumors, chronic inflammatory disease, rheumatic autoimmune disease, or thyroid dysfunction (current hypo- or hyperthyroidism), liver and kidney diseases. Five milliliter peripheral blood was collected using EDTA-anticoagulant tubes from all the participants. The study was approved by the ethics committees of all the involved hospitals and each subject gave written informed consent to participate in the study.

### DNA extraction, SNP selection and genotyping

Genomic DNA was extracted from whole blood samples collected with EDTA using a standardized BloodPrep DNA Chemistry isolation kit (Applied Biosystems, Forester City, CA, USA). All DNA samples were assessed for quality and quantity using a Nanodrop 2000, and DNA electrophoresis prior to genotyping. Except for rs74577862, we also selected the potential functional TagSNPs using the RegulomeDB score system ranging from categories 1-4, together with SNAP. Finally, we got SNP rs74577862 and rs62292784 in our analyses. Genotyping was performed using the high resolution melting (HRM) analysis. For quality control, Additionally, 10 % of samples were randomly selected for genotyping in duplicates and the results demonstrated a 100% degree of concordance among the duplicate pairs.

### Site-directed mutagenesis and dual-luciferase reporter assay

Site-Directed Mutagenesis were conducted using the Phusion Site-Directed Mutagenesis Kit (Thermo Fisher) according to the instructions of the manufacturer. The site mutagenesis was proved through sequencing. Then these two target sequences were cloned into pGL3-Basic vector (Promega, Madison, WI, USA), respectively. Negative control pGL3-Basic and reconstructed plasmids containing rs74577862 wild type or mutation type were respectively co-transfected into HAECs cell with pRL-SV40 vector, which expressed renilla luciferase as the transfection control, using Lipofectamine 3000 Reagent (Invitrogen, Waltham, MA, USA) after planting in 24-well plates for 24 h. luciferase activity was measured using the Dual-Luciferase Reporter Assay System (Promega) according to the manufacturer’s protocol, and all experiments were performed three times in triplicate.

### Quantification of gene expression using real-time PCR

Expression of the ADIPOQ gene in the plaque tissues of 50 individuals was confirmed by real time PCR. Assays were performed using TaqMan gene expression probes and reagents (Life Technologies) and run on a 7900HT Real Time PCR System (Applied Biosystems). GAPDH was used as the reference gene.

### Statistical analysis

Pearson’s χ2-test, or t-test was adopted to examine the differences in demographic variables and distributions of genotypes between cases and controls, when appropriate. Significant departure of genotype frequencies from Hardy-Weinberg expectation and test of differences in allele frequencies between cases and controls were determined by the chi-square test. Multivariate logistic regression analyses were used and expressed in terms of adjusted odds ratio (OR) and 95% confidence interval (CI) with adjustment for age, gender, smoking status, and drinking status. Multiple testing were conducted using Bonferroni correction, and 0.025 was used as the p value threshold for the two variants. Statistical analyses were undertaken using STATA (version 13.1, StataCorp LP, TX, USA).
